# Supplementary material for: Multienzyme deep learning models improve peptide de novo sequencing by mass spectrometry proteomics
Source: PLoS Comput Biol. 2023 Jan 20;19(1):e1010457. doi: 10.1371/journal.pcbi.1010457 (PMC9891523; doi:10.1371/journal.pcbi.1010457)
Supplement: S1 Text — A. Comparing all SEM models against the Kilo MEM model; B. Generalizability as a function of the number of SEM-combined training sets. These results show the same trends as the ones obtained on the Detroit test set. (DOCX) [file pcbi.1010457.s001.docx]

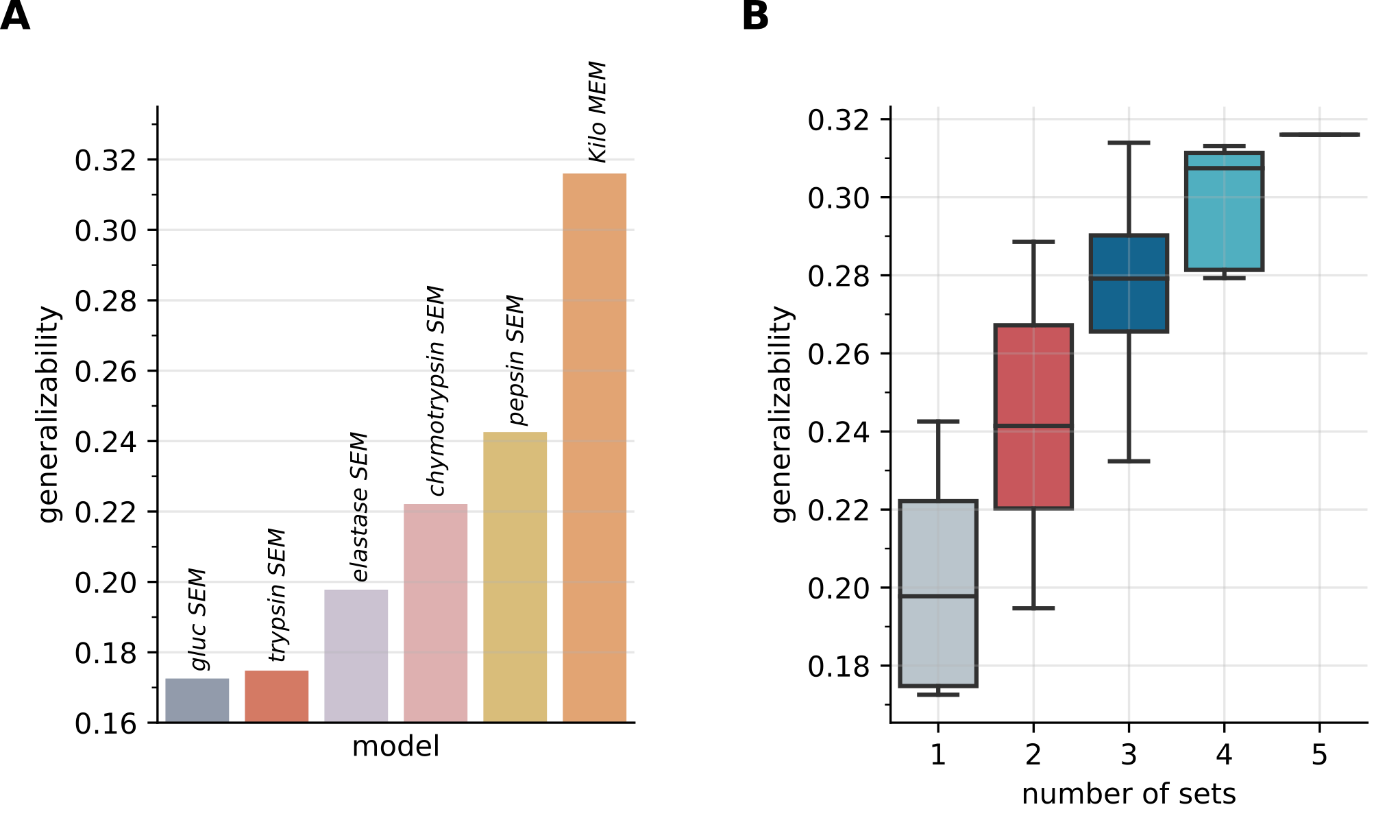


**S1 Supplementary Information.**Singel Enzyme (SEM) and Multi Enzyme (MEM) models performance on the Giga test set.  **A.**Comparing all SEM models against the Kilo MEM model; **B.**Generalizability as a function of the number of SEM-combined training sets. These results show the same trends as the ones obtained on the Detroit test set.
