# Supplementary material for: Multienzyme deep learning models improve peptide de novo sequencing by mass spectrometry proteomics
Source: PLoS Comput Biol. 2023 Jan 20;19(1):e1010457. doi: 10.1371/journal.pcbi.1010457 (PMC9891523; doi:10.1371/journal.pcbi.1010457)
Supplement: S2 Text — A. Number of matching peptides extracted from each sample type across all models; B. Number of extracted peptides for each deep learning model; C. Length distribution for the mAbs matching peptides across all considered models. The color scheme for the samples and models is at the bottom. (DOCX) [file pcbi.1010457.s002.docx]

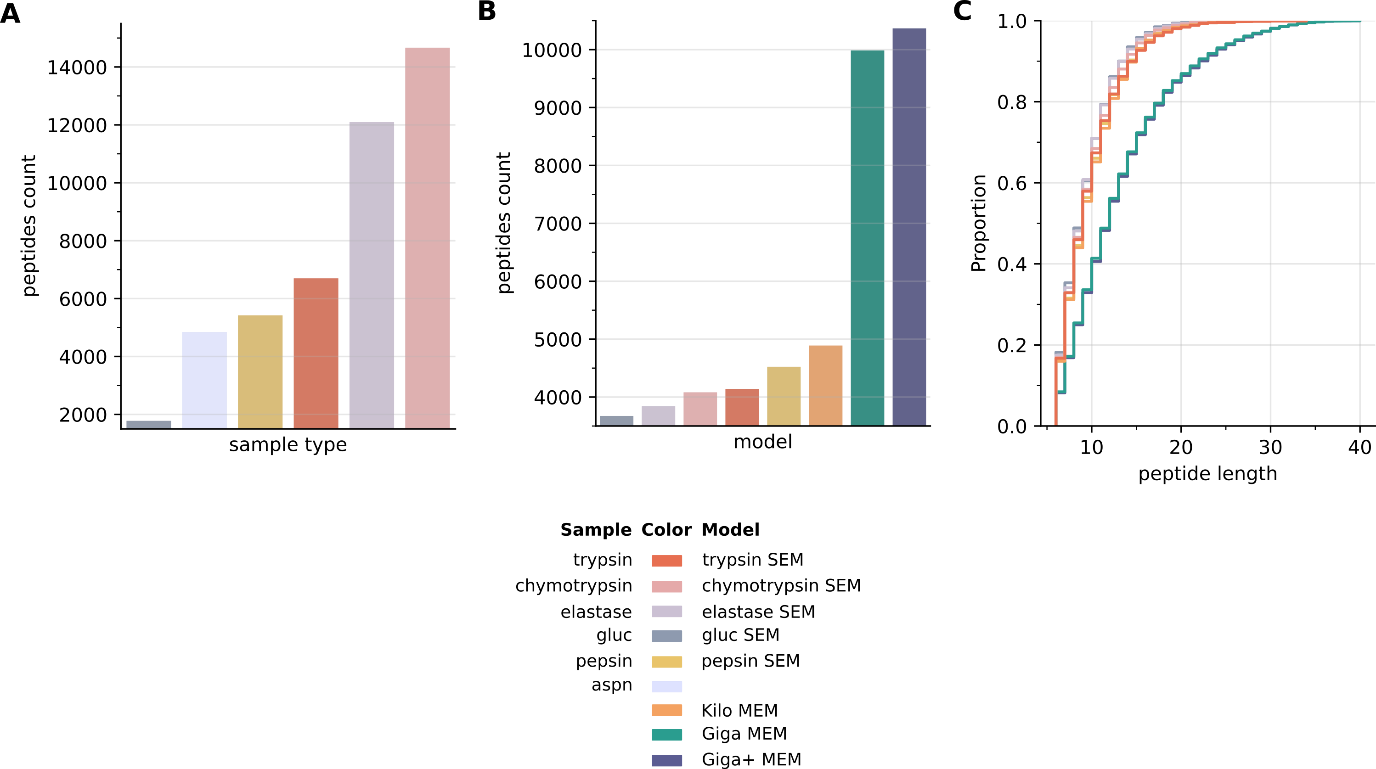


**S2 Supplementary Information.**De novo performance summary from the commercial monoclonal antibodies (mAbs) samples **A.**Number of matching peptides extracted from each sample type across all models; **B.**Number of extracted peptides for each deep learning model; **C.**Length distribution for the mAbs matching peptides across all considered models. The color scheme for the samples and models is at the bottom.

De novo decoded MS data show that the mAbs samples digested with chymotrypsin and elastase produced more easy-to-decode MS spectra than the remaining sample types. On the other hand, Giga+ and Giga MEM models were superior to the rest of the deep learning models, extracting over 10000 confirming peptides. Furthermore, they also extracted more peptides across a wide range of peptide lengths.
