# Supplementary material for: Multienzyme deep learning models improve peptide de novo sequencing by mass spectrometry proteomics
Source: PLoS Comput Biol. 2023 Jan 20;19(1):e1010457. doi: 10.1371/journal.pcbi.1010457 (PMC9891523; doi:10.1371/journal.pcbi.1010457)
Supplement: S3 Text — A. Positional frequency values across all residues composing the ten mAb polypeptides proteins; B. Positional frequency information for the heavy and light subunits; C. Confident positional score (CS) values for the variable and constant domains of the heavy and light chains. For the color scheme, we used dark and light brown colors for the heavy and light chains, respectively. (DOCX) [file pcbi.1010457.s003.docx]

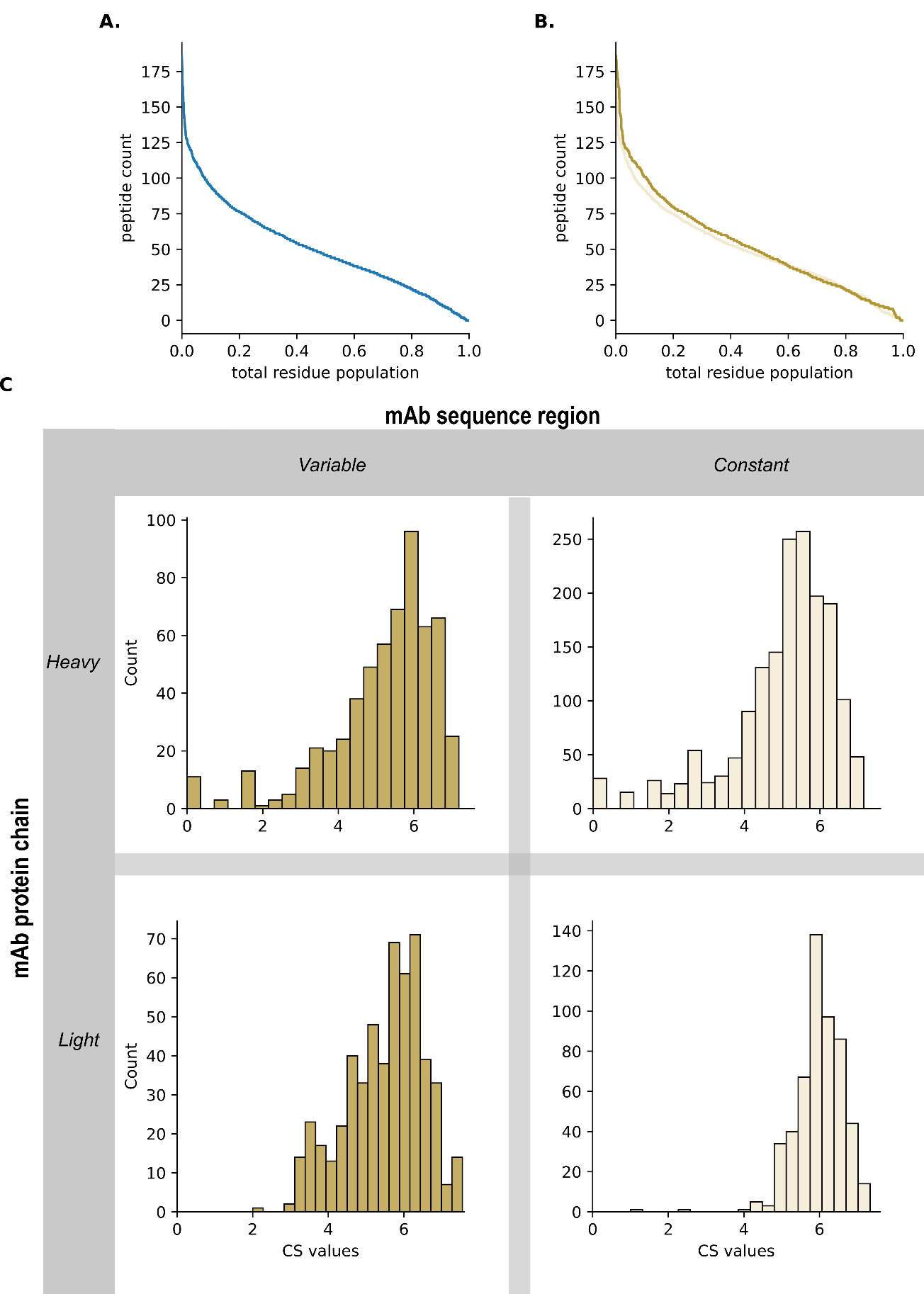


**S3 Supplementary Information.**Positional mAb data for the Giga+ MEM model de novo results: **A.**Positional frequency values across all residues composing the ten mAb polypeptides proteins; **B.**Positional frequency information for the heavy and light subunits; **C.**Confident positional score (CS) values for the variable and constant domains of the heavy and light chains. For the color scheme, we used dark and light brown colors for the heavy and light chains, respectively.
